# Genome sequence, transcriptome, and annotation of rodent malaria parasite *Plasmodium yoelii nigeriensis* N67

**DOI:** 10.1186/s12864-021-07555-9

**Published:** 2021-04-26

**Authors:** Cui Zhang, Cihan Oguz, Sue Huse, Lu Xia, Jian Wu, Yu-Chih Peng, Margaret Smith, Jack Chen, Carole A. Long, Justin Lack, Xin-zhuan Su

**Affiliations:** 1 Malaria Functional Genomics Section, Laboratory of Malaria and Vector Research, National Institute of Allergy and Infectious Disease, National Institutes of Health, Bethesda, MD 20892-8132 USA; 2 NIAID Collaborative Bioinformatics Resource (NCBR), Frederick National Laboratory for Cancer Research, Leidos Biomedical Research, Inc., Frederick, MD 21702 USA; 3 Advanced Biomedical Computational Science, Frederick National Laboratory for Cancer Research, Leidos Biomedical Research, Inc., Frederick, MD 21701 USA; 4 State Key Laboratory of Medical Genetics, Xiangya School of Medicine, Central South University, Changsha, Hunan 410078 People’s Republic of China; 5 The NCI sequencing facility, 8560 Progress Drive, Room 3007, Frederick, MD 21701 USA

**Keywords:** *Plasmodium*, Mouse, DNA sequence, Transcript, Proteome, Polymorphism

## Abstract

**Background:**

Rodent malaria parasites are important models for studying host-malaria parasite interactions such as host immune response, mechanisms of parasite evasion of host killing, and vaccine development. One of the rodent malaria parasites is *Plasmodium yoelii*, and multiple *P. yoelii* strains or subspecies that cause different disease phenotypes have been widely employed in various studies. The genomes and transcriptomes of several *P. yoelii* strains have been analyzed and annotated, including the lethal strains of *P. y. yoelii* YM (or 17XL) and non-lethal strains of *P. y. yoelii* 17XNL/17X. Genomic DNA sequences and cDNA reads from another subspecies *P. y. nigeriensis* N67 have been reported for studies of genetic polymorphisms and parasite response to drugs, but its genome has not been assembled and annotated.

**Results:**

We performed genome sequencing of the N67 parasite using the PacBio long-read sequencing technology, de novo assembled its genome and transcriptome, and predicted 5383 genes with high overall annotation quality. Comparison of the annotated genome of the N67 parasite with those of YM and 17X parasites revealed a set of genes with N67-specific orthology, expansion of gene families, particularly the homologs of the *Plasmodium chabaudi* erythrocyte membrane antigen, large numbers of SNPs and indels, and proteins predicted to interact with host immune responses based on their functional domains.

**Conclusions:**

The genomes of N67 and 17X parasites are highly diverse, having approximately one polymorphic site per 50 base pairs of DNA. The annotated N67 genome and transcriptome provide searchable databases for fast retrieval of genes and proteins, which will greatly facilitate our efforts in studying the parasite biology and gene function and in developing effective control measures against malaria.

**Supplementary Information:**

The online version contains supplementary material available at 10.1186/s12864-021-07555-9.

## Background

Malaria is one of the deadly tropical infectious diseases that impacts the health of hundreds of millions of people [1]. The lack of an effective vaccine, emergence of drug resistant parasites and insecticide resistant mosquitoes, and incomplete understanding of the disease mechanisms are the major factors that impede disease control and elimination. Vaccine development and in-depth studies of disease molecular mechanisms using human populations are limited by ethical regulations and relatively high costs. Animal disease models such as parasites infecting rodents and non-human primates are important systems for studying malaria and have been widely used for vaccine development and for studying the molecular mechanisms of host-parasite interaction [2, 3]. Of course, results obtained from animal models need to be verified in human infection because there are differences in disease mechanism due to variation in genetic backgrounds of both the parasites and the hosts.

*Plasmodium yoelii* is one of the rodent malaria species that includes several parasite strains or subspecies well-characterized genetically and phenotypically [4, 5]. Some of the *P. yoelii* strains are genetically diverse, whereas others are closely related or derived from a common ancestor during laboratory passages in mice [4, 6, 7]. Mice infected with these *P. yoelii* strains generally have dramatic differences in parasitemia, disease severity, pathology, and host immune response [8]. For example, *P. y. yoelii* 17X (or 17XNL) and *P. y. yoelii* 17XL (or YM) are closely related parasites genetically. Indeed, these parasites were derived from a parasite isolated from a wild thicket rat in the Central African Republic [7]. 17XL and YM lines became fast growing and lethal during passages of 17X parasites in mice in two separate laboratories, whereas 17X or 17XNL remained slow growing and non-lethal [7]. These parasites also stimulate different host responses and pathology [9–12]. Another example of parasites having closely related genomes but with different virulence is the *P. y. nigeriensis* N67 and *P. y. nigeriensis* N67C parasite pair. The N67 is also a natural parasite of thicket rat (*Thamnomys rutilans*) in Western Nigeria [13]. The genomes of N67 and N67C are very similar [5, 6]; however, they produce quite different disease phenotypes in C57BL/6 mice. Infection of N67 stimulates a strong early type I interferon (IFN-I) response, leading to a decline of parasitemia to below 5% day 7 post infection (pi). The parasitemia rebounds to 50–60%, and the host dies at day 20 pi [14]. In contrast, mice infected with N67C produce a strong T cell and INF-γ mediated inflammatory responses and die day 7 pi [15]. A C741Y amino acid substitution in the *P. yoelii* erythrocyte binding-like protein (PyEBL) contributes to the differences in virulence and immune response, but other parasite genes also play a role in the differences in disease phenotypes [16]. Identification of the genes or genetic differences between N67 and N67C parasite will facilitate our understanding of the molecular mechanisms of virulence and disease phenotypes in these infections.

With the advance of DNA sequencing technologies, the genomes and transcriptomes of many rodent malaria parasites, including those of YM, 17X, and 17XNL strains, have been sequenced and annotated [17–21]. The genomes of *Plasmodium berghei* and *Plasmodium chabaudi* parasites are approximately 18.5–19 Mb, whereas the *P. yoelii* YM genome is 22.75 Mb containing 5675 predicted genes [19]. There are only eight genes with single nucleotide polymorphisms (SNPs) detected between the genomes of the YM and 17X strains [19], supporting isogenic parasites recently derived from the same ancestor [7]. Although RNA and DNA sequencing studies using short Illumina reads from the N67 parasite have been previously carried out to investigate genetic polymorphisms and parasite response to drugs [6, 20], the N67 genome has not been assembled and annotated, which impedes studies of the gene functions, parasite biology, and virulence of the parasite. In this study, we sequenced the genome of the N67 parasite using PacBio sequencing technology that produces long sequence reads, assembled, and annotated its genome based on de novo assembled genome sequences and multiple transcriptomes. Comparison of the N67 genome sequences with those of the YM and 17X parasites revealed a set of proteins with N67-specific orthology, protein families predicted to regulate host immune responses, expansion of critical gene families, and a large number of SNPs and indels that pass stringent filtering criteria. These results have the potential to greatly facilitate our efforts in studying the parasite biology and in developing effective control measures against malaria.

## Results

### Genome sequencing, read statistics, and de novo assembly of the parasite genomes

We prepared DNA samples for PacBio sequencing from the N67 parasite we obtained previously [5]. Genomic DNAs were fragmented and sequenced on a PacBio Sequel using PacBio SMRT cell long read technology [22]. The polymerase reads from sequencing machine were first filtered to remove barcodes and low-quality sequences using the Hierarchical Genome Assembly Process (HGAP) (Fig. S1a). We obtained 1,111,721 subreads consisting of 6,733,837,360 bp for the N67 parasite, providing 233 mean coverages with an averaged barcode quality of 72. The longest subread length was 195,628 bp and the mean read length was 70,695 bp. The subreads were then assembled into 61,130 circular consensus sequencing (CCS) reads with a mean CCS coverage of 13.5-fold for the parasite.

We next de novo assembled the N67 CCS reads into 121 contigs consisting of 21,277,183 bp, with the largest contig being 979,279 bp (Table 1). For the assembled sequences, the N50 index was 300,848 bp with 95.8% of the N67 sequences in contigs > 50 kb (Fig. S1b). The GC content of the sequences for the parasites is ~ 22% for the nuclear genome and ~ 30% for mitochondrial and the plastid genomes, similar to those of the 17X parasite.

**Table 1:** *Plasmodium yoelii nigeriensis* N67 genome assembly statistics using Hierarchical Genome Assembly Process (HGAP)

Assembly Statistics	N67
Number of Contigs:	121
Number of chromosomes in the reference (17X):	16
Number of assembly bases:	21,277,183
Number of reference bases:	23,083,521
Number of LCBs:	11
Number of Blocks:	214
Breakpoint Distance:	204
DCJ Distance:	19
SCJ Distance:	408
Number of Gaps in Reference:	35,690
Number of Gaps in Assembly:	31,011
Number of missing chromosomes:	2
Number of extra contigs:	20
Number of Shared Boundaries:	0
Number of Inter-LCB Boundaries:	9
Contig N50:	300,848
Contig N90:	5956
Min contig length:	5956
Max contig length:	979,279
NG50	290,851
NA50	193,872
G + C content (%)	21.72

Descriptions of the blocks of alignments and statistics identified by Mauve: LCB is defined as a set of local alignments that occur in the same order and orientation (free from internal rearrangement) in a pair of genomes. SCJ (Single-Cut-or-Join) and DCJ (double-cut-and-join) distances are rearrangement metrics that measure the minimum number of cut or join operations needed to transform one genome into another, whereas breakpoint distance is the number of non-conserved adjacencies.

GC (%) is the total number of G and C nucleotides in the assembly, divided by the total length of the assembly.

N50 is the length for which the collection of all contigs of that length or longer covers at least half (90% for N90) of the assembly.

NG50 is the length for which the collection of all contigs of that length or longer covers at least half the reference genome.

NA50 is similar to N50 (corresponding metric without “A”), based on aligned blocks instead of contigs

### Alignment of N67 sequences to the 17X assembled genome

**Fig. 1:**
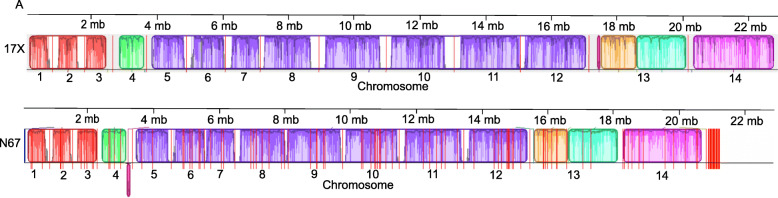
Alignment of Hierarchical Genome Assembly Process (HGAP) assembled N67 contigs to the 17X chromosomes. The alignments were generated using progressiveMauve. Each color corresponds to a localized co-linear block (LCB) that is conserved across the two genomes. Inside each LCB, the jagged dark lines represent the similarity profile; with darker colors representing higher similarity regions. The vertical red lines indicate chromosome boundaries in 17X and the contig boundaries on the N67 sequences. Note a contig on N67 chromosome 4 that is inverted (presented under the chromosome line) in reference to that of the 17X sequence

Before investigating the diversity of the N67 genome and performing genome annotation, we aligned both the CCS reads and the assembled contigs to the updated 17X reference genome in PlasmoDB, version 46 (https://plasmodb.org/plasmo/) [18, 22] using Minimap2 [23] and the progressiveMauve algorithm [24] that performs contig-by-contig alignment between the assembly and the 17X reference (Fig. S1a). A total of ~ 23 Mb from the N67 CCS reads were aligned to the 14 chromosomes of the 17X parasite, 34,324 bp to the plastid genome, and 6083 bp to the mitochondrial genome, suggesting good overall genome coverages (Table 2). The mean CCS read coverages were 11.0–13.7 for the autosomes, 43.9 for the plastid genome, and 334.3 for the mitochondrial genome. In addition to the base-level alignment, we also aligned 101 of the 121 N67 contigs to the 17X reference genome using the progressiveMauve algorithm (Fig. 1) and 18.1 Mb of the 21.1 Mb (86%) de novo assembled N67 genome to the 17X genome using Minimap2. The low GC content of the parasite DNA and the abundance of low-complexity repeats in the genomes pose challenges to the assembly process and the alignment of the assembled N67 genome to the 17X reference. Therefore, approximately 14% of the N67 assembly did not align to the 17X genome.

**Table 2:** Chromosomal lengths and mean coverages of *Plasmodium y. nigeriensis* N67 parasite

Name	Initial alignments		
	Length (bp)	Mapped (bp)	Mean cov. (SD)
Py17X_01_v3	815,147	10,290,387	12.6 (6.8)
Py17X_02_v3	982,731	10,831,889	11.0 (7.0)
Py17X_03_v3	869,676	10,685,579	12.3 (6.9)
Py17X_04_v3	1,021,539	13,954,592	13.7 (6.6)
Py17X_05_v3	1,210,876	16,468,806	13.6 (7.9)
Py17X_06_v3	1,185,181	16,394,574	13.8 (8.1)
Py17X_07_v3	1,064,364	14,157,540	13.3 (9.9)
Py17X_08_v3	1,791,361	23,570,622	13.2 (7.5)
Py17X_09_v3	2,046,250	27,675,754	13.5 (9.2)
Py17X_10_v3	2,065,729	28,355,490	13.7 (9.2)
Py17X_11_v3	2,012,183	26,754,907	13.3 (5.7)
Py17X_12_v3	2,085,115	27,989,086	13.4 (5.8)
Py17X_13_v3	3,033,250	41,535,459	13.7 (7.7)
Py17X_14_v3	2,859,712	38,462,510	13.5 (5.9)
Py17X_API_v3	34,324	1,508,111	43.9 (6.1)
Py17X_MIT_v3	6083	2,033,752	3,34.3 (45.5)
Total	23,043,114	307,127,195	13.1

### RNA-Seq data and de novo transcriptome assembly of the N67 parasite

To facilitate genome annotation and gene prediction, we also sequenced mRNA of blood stages from eight mice infected with N67 using Illumina sequencing method. Overall, 82.9% of the RNA-Seq reads from the samples uniquely mapped to the 17X genome and were retained for transcriptome assembly, The majority of the remaining reads were either uniquely mapped to the mouse genome (4.7%) or did not map to any of the human, mouse, bacteria, fungi and virus genomes (9.3%) based on results from the FastQ Screen [25]. The remaining 3.1% of reads were mapped to human, fungi, bacteria or multiple genomes. We then used Trinity [26] to perform de novo transcriptome assembly and obtained 25,689 transcripts containing 39,856,633 bp with an average GC content of 23.5% (Table S1**)**. The N50 was 1952 bp with the largest transcript being 19,550 bp. The N67 Illumina reads were aligned to the de novo assembled *P. yoelii* transcriptome using Bowtie2 [27], resulting in 95.4% of the N67 read pairs concordantly aligned to the assembled transcriptome, showing a high level of overall read support for the assembly.

### Gene predictions and functional annotation

We predicted 5383 genes/proteins from the N67 genome, including all the sequences not aligned to the 17X genome, using the MAKER pipeline [28] as described in the Methods (Table S2). For a high quality and well-annotated assembly, at least 90% of the predicted proteins are required to have annotation edit distance (AED) values of less than 0.5 [28]. For the N67 proteome, 98 and 94% had AED (base pair level) and eAED (exon level) values less than 0.5, respectively. Additionally, more than 50% of the proteome should ideally contain a recognizable protein domain for a well-annotated proteome [28]. Ninety-two percent of the predicted N67 proteins have recognizable domains and/or are assigned to protein families. Furthermore, the smallest predicted N67 protein has 16 amino acids (N67_005372, Table S2), similar to the smallest predicted protein of 15 amino acids in the 17X proteome. Search of N67_005372 protein sequence (MRVNKYVSVNMKMNYT) against the 17X and YM proteome did not return any hit; however, it has a 79% sequence identity to serine hydroxymethyltransferase of thermoacidophilic archaea *Thermoplasma volcanium*.

Search of the N67 proteome against InterPro database (https://www.ebi.ac.uk/interpro/search/sequence/) of protein families, domains and functional sites using InterProScan revealed that the largest five groups of proteins were the YIR antigens (750 members), P-loop containing nucleoside triphosphate hydrolases (272), subtelomeric PYST-A proteins (118), WD40-repeat-containing domain superfamily (89), and homologous proteins of *P. chabaudi* erythrocyte membrane protein 1 (PcEMA1) (83) (Table S3). One single copy of the PcEMA1 gene was identified in IP, CB, DK, KA, and DS strains of *P. chabaudi* earlier [29], and there is one PcEMA1 homolog in the 17X and YM parasites as well as in *P. berghei* ANKA [19]. Interestingly, 13 copies of PcEMA1 genes were detected in the *P. chabaudi* AS strain [19]. The PcEMA1 was initially described from *P. chabaudi* parasites as an acidic phosphoprotein that might modulate the structure of the red cell membrane to the advantage of the parasite [30]. It has two tandem repats (16 × 8 AA and 2 × 9 AA) that may mediate genetic recombination and gene member expansion possibly through microhomology-mediated end joining (MMEJ) [31]. The expansion of this gene family in N67 parasites suggests that the PcEMA1 proteins may play a role in interaction with host immune system. Some other interesting groups included 43 proteins with DEAD/DEAH box helicase domain, 22 proteins with AP2/ERF domain, and 7 proteins with Rh5 coiled-coil domain.

We also searched the predicted N67 proteome for protein domains associated with pathways within the Reactome pathway database. The top five largest Reactome groups were major pathways of rRNA processing (122 proteins), regulation of expression of SLITs and ROBOs (117), SRP-dependent cotranslational protein targeting to membrane (92), GTP hydrolysis and joining of the 60S ribosomal subunit (91), and L13a-mediated translational silencing of ceruloplasmin expression (89) (Table S4). Interestingly, there were also many proteins involved in viral mRNA translation (78) and immune responses such as pathways of antigen processing (64), neutrophil degranulation (36), NFκB activation in B cells (35), CLEC7A (Dectin-1) signaling (35), downstream TCR signaling (35), FCERI mediated NFκB activation (35), interleukin-1 signaling (35), NIK noncanonical NFκB signaling (35), Vpu mediated degradation of CD4 (35), TNFR2 non-canonical NFκB (34), and genes in MHC class II antigen presentation (23) (Table S5). The molecules in the viral mRNA translation are mostly structural constituents of ribosome proteins that are likely essential for the translation of parasite proteins. *Toxoplasma* parasites secrete effector proteins into the host cell to co-opt host transcription factors and modulate host immune responses [32]. Some of the proteins grouped with immune response pathways could play important roles in regulating host immune response to infection of liver stages that invade nucleated host cells.

### Estimates of completeness of the N67 genome and transcriptome

We next used Benchmarking Universal Single-Copy Orthologs (BUSCO 3.0.2) to assess the completeness of the assembled N67 genome. Of the 3642 *Plasmodium* and 446 *Apicomplexa* BUSCO gene sets, 3369 (92.5%) and 431 (96.6%) were present in the N67 genome assembly, respectively (Table S6). We also evaluated the extent to which the assembled N67 transcriptome matched the BUSCO gene sets across the *Apicomplexa* and *Plasmodium*. Approximately 92.8% of the BUSCO *Apicomplexa* gene set and 71.3% of the *Plasmodium* gene set were present in the assembled N67 transcriptome (Table S6). The N67 transcriptome genes matching the *Plasmodium* BUSCO gene set included 1448 complete and single-copy genes (39.8%), 1149 (31.5%) complete and duplicated genes, and 338 fragmented sequences (9.3%) (Table S6). There were also 707 genes (19.4%) missing from the *Plasmodium* BUSCO gene set; some of the missed genes might not be expressed in the blood stages. The long reads from PacBio sequencing appear to provide more complete gene assembly than those from short Illumina reads. 

### *P. yoelii* common orthogroups and putative proteins with N67-specific orthology

**Fig. 2:**
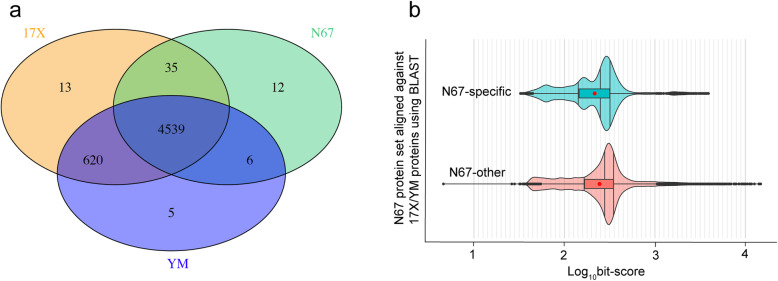
Shared and strain-specific orthogroup counts identified from *Plasmodium y. yoelii* YM, *P. y. yoelii* 17X, and *P. y. nigeriensis* N67 parasites using OrthoFinder [33]. **a** Venn diagram of shared and strain-specific orthogroups; **b** log_10_-transformed bit-score distributions for N67 proteins that are not assigned to any orthogroup plus those in N67-specific orthogroups (N67-specific) and proteins assigned to orthogroups having at least one 17X or YM protein (N67-other). The bit-scores are derived from pairwise BLAST alignments within the Orthofinder framework, where all queries were N67 protein sequences that were aligned against the 17X and YM sequences. The red dots indicate the mean values of bit-score distributions, whereas the vertical lines within the violins indicate the median, upper and lower quartile values

The N67 and 17X (or YM) parasites belong to two subspecies of *P. yoelii*, and the genomes of these parasites are quite diverse [4, 6]. It is potentially interesting to identify genes common and unique (or highly diverse) in these parasite genomes. Therefore, we compared the 5383 predicted proteins from N67 with 6092 17X proteins and 5685 YM proteins using OrthoFinder [33] and identified a core set of 4539 orthogroups shared among the N67, 17X, and YM genomes (Fig. 2a). Out of a total 17,160 proteins from the three parasite strains, 17,035 (99.3%) were placed in 5230 orthogroups based on searches of sequence similarity using DIAMOND within the latest OrthoFinder framework [34, 35]. Of the 5383 N67 proteins, 5294 were assigned to orthogroups, including 110 in 12 N67 specific orthogroups (Table S7 and Table S8**)**. There were also 89 proteins that could not be assigned to any orthogroup, leading to a total of 199 proteins that appear to have N67-specific orthology. These proteins had slightly lower pairwise bit-scores than those assigned to the orthogroups with at least one 17X or YM protein (Fig. 2b).

To further characterize N67-specific proteins, we used BLAST to align the 199 N67 proteins against the 17X proteome and showed that the majority proteins had motifs matching members of highly diverse gene families. Among the 199 proteins, 91 are hypothetical or uncharacterized proteins, 64 are PIR/YIR proteins, 22 are Fam-A/B proteins, and five are reticulocyte binding proteins (Table S9). Clustering the proteins based on sequence similarity generated three dendrograms, one consisting of Fam-A and Fam-B proteins (Fig. S2a and Table S9), another one consisting of 10 YIR proteins (Fig. S2b), and a third one of three subclusters of YIR proteins (Fig. S2c). The YIR proteins in cluster B and C are quite different and could not be clustered together, suggesting potentially different origins.

### Gene families from three *P. yoelii* parasites

**Fig. 3:**
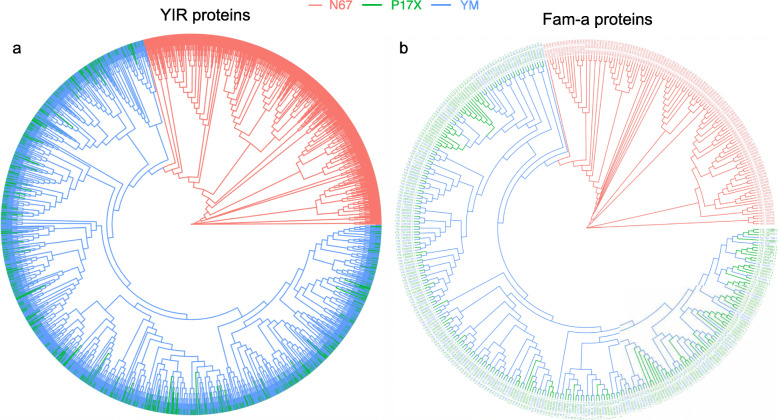
Clustering of YIR and Fam-A proteins from the *Plasmodium y. nigeriensis* N67, *P. y. yoelii* 17X, and *P. y. yoelii* YM parasites. The predicted protein sequences were aligned using ClustalW algorithm in msa R package, and the dendrograms were inferred and visualized using the ape, seqinr, and ggtree packages in R. **a** YIR proteins from N67, 17X, and YM parasites; **b** Fam-A proteins. Proteins are colored based on their parasite origins

**Fig. 4:**
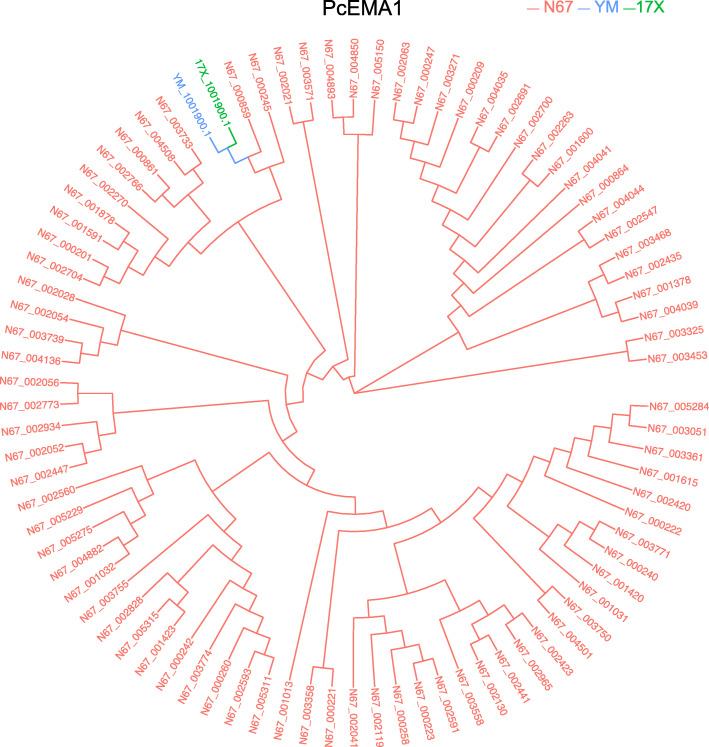
Clustering of homologous *P. chabaudi* erythrocyte membrane protein 1 (PcEMA1) from *Plasmodium y. nigeriensis* N67, *P. y. yoelii* 17X and *P. y. yoelii* YM parasites. The gene family is expanded only in the N67 parasite. The predicted protein sequences were aligned using ClustalW algorithm in msa R package, and the dendrogram was inferred and visualized using the ape, seqinr, and ggtree packages in R. Proteins are colored based on their parasite origins

Among the predicted genes and proteins, we identified 22 gene families that have been previously found in *Plasmodium yoelii* [36] with at least one member detected in N67 (Table S10). The gene families consist of 1475 genes (24% of the predicted genes) for 17X, 1141 genes (21%) for N67, and 1075 genes (19%) for YM parasite (Table S10). The largest gene families are the *yir* and *fam-a/b/c/d* families. There are 1057 *yir* and 301 *fam-a/b/c/d* genes for 17X, 750 *yir* and 213 *fam-a/b/c/d* genes for N67, and 773 *yir* and 190 *fam-a/b/c/d* genes for YM parasite, respectively. As expected, clustering of YIR and Fam-A protein families showed that the proteins from N67 grouped separately from those of 17X and YM (Fig. 3a and b), consistent with N67 being a subspecies of *P. yoelii*. The true numbers of *yir* and *fam-a/b/c/d* genes for the N67 and YM parasites could be larger because some genes in these gene families are likely not assembled into the genome. Other important gene families include genes encoding early transcribed membrane proteins (ETRAMPs), lysophospholipase, erythrocyte membrane antigens, and reticulocyte binding proteins associated with host-parasite interactions and Cys6 (6-Cysteine) proteins. As noted above, there are 83 copies of the gene encoding PcEMA1 homologs [30] in the N67 parasite, compared with only one gene in the 17X and YM parasites, respectively (Table S10). Clustering of the PcEMA1 proteins from the three parasites showed that N67_000859 and N67_000245 were closely related to the two single copies from 17X (17X_10019001) and YM (YM_100119001) (Fig. 4). Similarly, there are nine genes encoding haloacid dehalogenase-like hydrolase in N67, but only 4 genes in both 17X and YM parasites. In contrast, the number of genes encoding reticulocyte binding proteins appears to be reduced in N67 parasites; 12 genes for N67 (including five from N67 specific orthogroups), whereas 17X and YM have 33 and 31, respectively. The expansion of the PcEMA1 homolog genes deserve additional investigation.

### Sequence polymorphisms between N67 and 17X

Initial alignment of the N67 sequences to those of 17X identified 486,102 SNPs and 41,317 indels, leading to approximately one SNP per 47.3 bp and one indel per 556.7 bp DNA between 17X and N67 assuming a genome size of 23 Mb [19]. The number of SNPs of this study is similar to the previous 458,922 SNPs from Illumina reads [6]. We further filtered the SNPs using Ensembl Variant Effect Predictor (VEP) based on the impact of the variants on the protein function and the following criteria: coverage in both strains with a minimum depth of 5X and a dominant allele frequency of 75%. We identified 69,413 SNPs and 11,076 indels that passed the following criteria and were predicted to have high or moderate impacts (Table S11). These variants represent approximately one SNP per 331.4 bp and one indel per 2076.6 bp of DNA. High impact variants are those assumed to be disruptive on the protein functions such as total loss of function caused by protein truncation. Moderate impact variants are non-disruptive variants that might change protein effectiveness such as missense mutations, in-frame indels, and splice-region variants outside the canonical splice site (http://uswest.ensembl.org/info/genome/variation/prediction/predicted_data.html). Given that there are 80,489 and 79,946 high or moderate impact variants between N67 vs 17X and YM, respectively (Table S11), it is quite interesting that with so many differences between the genomes, the YM (or 17XNL) and N67 parasites could be genetically crossed without apparent difficulty in terms of mating or genetic crossover [5, 37].

## Discussion

This study reports an annotated genome for the N67 parasite, a subspecies of *P. yoelii nigeriensis*, using PacBio long sequence reads and RNA-Seq sequences from blood stage parasites. The genomes of several *P. yoelii* strains belonging to different subspecies (*P. y. yoelii*), including 17X, 17XNL, and YM strains, have been reported and well-characterized [17, 19, 21]. Although Illumina-based RNA and DNA sequencing of the N67 parasite have been performed for studying genetic polymorphisms and parasite response to drugs [6, 20], assembly of the N67 genome and prediction of gene functions have not been reported previously due to difficulties in assembling AT-rich short sequence reads. Our study predicted and annotated 5383 genes for N67 and identified a set of proteins with N67-specific orthology. Approximately 14% of the assembled N67 sequences did not align to the 17X genome. The unaligned sequences were likely due to high level of sequence diversity between the 17X and N67 genomes and the limitations of the assembling process. There are more than 80,000 SNPs and indels having medium to high functional impacts between the N67 and 17X genomes. Improvement of the N67 genome with increased overall alignment with the 17X reference would likely increase the numbers of SNPs and indels. Interestingly, despite having highly diverse genomes (the level of diversity is greater than human and chimpanzee), the N67 and YM or 17XNL can be genetically crossed to produce progenies for mapping parasite and host genes [5, 37, 38]. The genome and transcriptome sequences and annotations of the N67 parasite we present here will be valuable resources for future studies on gene functions of this important *P. yoelii* subspecies.

The majority of studies using *P. yoelii* parasites involved the lethal YM (or 17XL) and nonlethal 17X (17XNL) strains. However, *P. y. nigeriensis* parasites have also been used as models for studying unique disease phenotypes, transmission in mosquitoes, strain-specific host immune responses and drug resistances [14, 15, 39–42]. The N67 and N67C are two isogenic strains of *P. y. nigeriensis* subspecies that cause very unique and interesting disease phenotypes in C57BL/6 mice. Whereas mice infected with N67 parasites produce an early peak of IFN-I that has been linked to suppression of parasitemia day 6 pi, mice infected with N67C produce low levels of IFN-I and die approximately day 7 pi due to T cell and IFN-γ mediated inflammation [14, 15]. A mutation (C741Y) in PyEBL partially contributes to the difference in disease phenotypes [16]; however, other genes in the genome are likely involved, particularly the variant gene families such as the *yir* and *fam-a/b/c/d* genes. For example, a locus at one end of chromosome 13 was shown to be significantly linked to many host genes functioning in IFN-I response pathways or interferon-stimulated genes (ISGs) [38]. Among the proteins in Reactome pathways that may play a role in viral mRNA translation and immune responses, several proteins can bind RNA and DNA or have endopeptidase activities. These proteins may function to activate NFκB signaling, ISG15 antiviral mechanism, and DDX58/IFIH1-mediated induction of IFN-α/β if they are secreted into the host cytoplasm, particularly in liver cells. Further experiments are required to demonstrate whether any of these proteins can influence host immune responses.

Approximately 20% of the annotated genes in the *P. yoelii* genomes are members of multi-copy gene families. The number of the *yir* genes in 17X is much larger than that of N67 or YM. Although the observation of more *yir* genes in the 17X genome could be due to more complete and better annotated 17X genome, it is not surprising to find variation in the number of *yir* genes. The *yir* genes belongs to the *Plasmodium* interspersed repeat (*pir*) gene families. The *pir* genes are mostly distributed in the subtelomeric regions of chromosomes with gene copies numbering from a few dozen to hundreds or even over a thousand [43], and up to 40% of the *cir* gene (gene family from *P. chanbaudi*) repertoire are expressed during the intraerythrocytic cycle [44]. Many of the *pir* genes are expressed on the surface of iRBCs and merozoites and play an important role in immune evasion [45, 46]. In addition to the *yir* genes, the variation in number of genes encoding PcEMA1 homologs, haloacid dehalogenase-like hydrolases, reticulocyte binding proteins between N67 and 17X/YM are interesting, particularly the expansion of the gene encoding PcEMA1 homologs. These proteins likely play some important roles in host-parasite interaction and parasite development.

The assembly and annotation of the *P. y. nigeriensis* N67 genome provide important resources for further delineation of gene functions, comparative genomics, and evolutionary analysis. For example, genes in a genetic locus can be quickly identified after a chromosome segment is linked to a specific phenotype through genetic mapping. Despite differences in growth dynamics and disease phenotypes, the high level of genome diversity with similar morphology and life-cycle between subspecies of *P. y. yoelii* and *P. y. nigeriensis* raises an interesting evolutionary question: How can the major biological characteristics be preserved with such diverse genome sequences, including a large number of SNPs predicted to have high functional impacts? Detailed comparison of genetic polymorphisms and functional characterization of individual genes from different parasite strains and subspecies may reveal critical information on gene function and genome evolution.

## Conclusions

The rodent parasite N67 is an important strain of *P. y. nigeriensis* subspecies for studying host immune responses and parasite biology. The lack of an assembled and annoated genome has impeded functional studies of the parasite, including genetic mapping determinants playing important roles in modulating host IFN-I responses. This study provides the first assembled and annotated N67 parasite genome , including prediction of 5383 genes, although there are still many gaps in the genome. Comparison of the annotated N67 parasite genome with those of YM and 17X parasites reveals a large numbers of SNPs and indels that may have functional impact on parasite development and biology. Additionally, unique N67 gene sets, expansion of gene families, and genes potentially regulating host immune responses are also identified. Although further efforts such as manual curation are necessary to completely assemble the genome sequences, the assembled and annotated N67 genome with over 5000 predicted genes from this study will greatly facilitate our investigations of the parasite biology and disease mechanisms.

## Methods

### Parasite and infection of mice

The N67 parasite was initially obtained from MR4-BEI (https://www.beiresources.org/About/MR4.aspx) and were described previously [5]. Inbred female C57BL/6 mice, aged 6–8 weeks old, were obtained from NIAID/Taconic repository. The procedures for infecting mice with the parasites were as reported previously [5, 8]. Parasitemia was monitored by microscopic examination of Giemsa-stained thin blood smears.

### DNA preparation for PacBio sequencing

Mice were injected *ip* with an inoculum containing 1 × 10^6^ infected red blood cells (iRBCs). Blood samples (200 μl) with approximately 30–40% parasitemia were collected on day 4 after injection. Infected red blood cells collected in 1 ml 0.15% sodium citrate/PBS buffer were pelleted at 2000 rpm for 5 min in an Eppendorf centrifuge, re-suspended in 1 ml of PBS, and passed through two consecutive NWF filters (Zhixing Bio, Bengbu, China) to remove the host white blood cells [20]. The flow-through cell suspension was washed in 800 μl PBS 3X through centrifugation at 3000 rpm for 3 min. The pellet was dissolved in 400 μl lysis buffer (100 mM NaCl, 10 mM Tris, 25 mM EDTA, pH 8.0, 0.5% SDS) containing 20 μl RNase (500 μg/ml) and 20 μl protease K (10 mg/ml) and incubated at 50 °C overnight. DNA was extracted using 400 μl phenol, chloroform, and isopropanol at a ratio of 25:24:1 and precipitated by adding 2 volume 100% ethanol overnight at − 20 °C. The sample was centrifuged at 13,000 rpm for 15 mins at 4 °C, and the DNA pellet was washed in 500 μl 70% ethanol twice before addition of 20 μl water. The quality of DNA was estimated on 1% agarose gel showing a typical high molecular weight band.

### Fragmentation of DNA and PacBio sequencing

A SMRTbell library was constructed using standard PacBio library preparation procedure (Pacific Biosciences, Menlo Park, CA, USA). The genomic DNA was fragmented with the majority of DNA fragments above 20 kb, then the DNA was carried into the first enzymatic reaction to remove single-stranded overhangs and tailed with an A-overhang. Ligation with T-overhang SMRTbell adapters was performed and the SMRTbell library was purified. The size and concentration of the final library were assessed.

Sequencing primer and Sequel DNA Polymerase were annealed and bound, respectively, to the SMRTbell library. The library was loaded on PacBio Sequel using diffusion loading. SMRT sequencing was performed on the Sequel System with Sequel Sequencing Kit 3.0, 1200 min movies. Quality control (QC) for raw reads (subreads) generated from the sequencer were performed by the default SMRT Link QC pipeline. Pass-filter reads were then used as input for the genome assembly.

### Genome sequence assembly

The genome was assembled using HGAP v4.0, a standard assembler from PacBio SMRTLink software (Pacific Biosciences, Menlo Park, CA, USA), Subreads longer than 6 kb were designated as “seed reads” and used as template sequences for preassembly/error correction. After assembly, two rounds of polishing were performed to increase the consensus sequence quality of the assembly, including aligning the PacBio data to the contigs and computing consensus using the Arrow consensus caller (SMRTLink).

### RNA-Seq, transcriptome assembly and gene predictions

To assemble the N67 transcriptome, we extracted RNA from mixed-stage iRBC samples of eight mice infected with N67 and performed Illumina sequencing as reported previously [16]. The resulting RNA-Seq reads were trimmed with Trimmomatic [47] to remove the adapter sequences, and the reads were mapped to the 17X genome using the STAR aligner [48] and disambiguate [49] to identify the reads that exclusively align to the assembled N67 genome. RNA-Seq reads from the samples uniquely mapped to the N67 genome were retained for the transcriptome assembly step that was performed using Trinity [26].

Gene predictions were generated using the MAKER pipeline [28]. Specifically, MAKER utilized BLAST to align the de novo assembled N67 transcriptome and 17X transcriptome to the de novo assembled N67 genome, polished these alignments using Exonerate in a splice-aware fashion, and implemented SNAP and Augustus hidden Markov models (HMMs) to generate ab initio gene models [50, 51]. Functional analysis and annotation was performed with InterProScan [52] after homology searches of over 15 databases including Pfam, ProSite, TIGRFAM, and PANTHER. Our final set of N67 genes/proteins only include those that are adequately supported by the assembled N67 genome/transcriptome and the 17X proteome. For each predicted N67 protein, we computed two AED values (AED/eAED: at the base pair and exon levels) to quantify how well each N67 protein is supported by these data sources [53].

### Estimates of completeness

We quantified the completeness of the de novo assembled N67 transcriptome, genome, and proteome using BUSCO [54] against the single-copy orthologs conserved among *Apicomplexa* (446 BUSCOs) and *Plasmodium* (3642 BUSCOs) from the OrthoDB v10.1 database [55].

### Identification of orthologs

We used the Orthofinder [35] framework for identifying the ortholog sets among the 17X, YM and N67 proteins. Orthofinder utilizes DIAMOND [34] for identifying sequence similarity and DendroBLAST [56] for gene tree inference.

### Gene family clustering

Hierarchical clustering analyses were performed using MEGAX [57]. Protein sequences of YIR, Fam-A as well as proteins from the N67-specific orthogroups and the ones not assigned to any orthogroup were aligned using the ClustalW algorithm. The maximum likelihood method and Jones-Taylor-Thornton (JTT) matrix-based model were used to construct cladograms from the aligned sequences [58].

## Supplementary Information

**Additional file 1: Figure S1.** Strategies of genome assembly and annotation, and plot of contig length distributions of the *Plasmodium y. yoelii* N67 parasite genome assembly. **a**, Diagram illustrating the processes of aligning N67 CCS reads (A) and contigs (B) to the 17X genomes. HGAP, Hierarchical genome assembly process; CCS read, circular consensus sequencing read. **b**, Plot of contig length distribution. The X-axis is percentage of the contigs with lengths (base pair) greater than the values indicated on the Y-axis. **Figure S2.** Clustering of protein sequences from the *Plasmodium y. nigeriensis* N67-specific orthogroups and those that are not assigned to any orthogroup. The predicted protein sequences were aligned using ClustalW algorithm and clustered using procedures described in the Methods section. **a**, Fam-A/B proteins; **b**, YIR proteins (group 1); **c**, YIR proteins (group 2). Only bootstrap values higher than 70% are shown.

**Additional file 2: Table S1.** Statistics of *de novo* transcriptome sequence assembly of Illumina paired end reads using Trinity.

**Additional file 3: Table S2.** Predicted gene and protein sequences from *Plasmodium y. nigeriensis* N67 parasite genome and transcriptome.

**Additional file 4: Table S3.** Predicted proteins of *Plasmodium y. nigeriensis* N67 matching IntrPro domains.

**Additional file 5: Table S4.** Predicted *Plasmodium y. nigeriensis* N67 proteins that match those in Reactome pathways.

**Additional file 6: Table S5.** Lists of *Plasmodium y. nigeriensis* N67 genes clustered into selected Reactome pathways.

**Additional file 7: Table S6.** Completeness statistics of the predicted *Plasmodium y. nigeriensis* N67 genome and transcriptome in matching. *Apicomplexan* and *Plasmodium* benchmarking universal single-copy orthologs (BUSCO) gene sets.

**Additional file 8: Table S7.** Summary statistics of genes and orthogroups from three *Plasmodium yoelii* parasites.

**Additional file 9: Table S8.** Individual orthogroups containing genes from three *Plasmodium yoelii* parasites (17X, N67, and YM).

**Additional file 10: Table S9.** Sequences and functional annotation of genes in *Plasmodium y. negirenesis* N67 specific orthogroups.

**Additional file 11: Table S10.** Gene families and copy numbers from three *Plasmodium yoelii* strains.

**Additional file 12: Table S11**. Single nucleotide substitutions and indels between *Plasmodium y. nigeriensis* N67 (ALT) and *P. y. yoelii* 17XNL (REF) or *P. y. yoelii* YM parasites with predicted medium and high functional impacts.

## Data Availability

This assembled N67 genome has been deposited to GenBank with an accession numberof JAEVLW000000000.

## Abbreviations

YM: *Plasmodium y. yoelii* YM
17X: *Plasmodium y. yoelii* 17X
17XNL: *Plasmodium y. yoelii* 17XNL
17XL: *Plasmodium y. yoelii* 17XL
N67: *Plasmodium y. nigeriensis* N67
IFN-I: Type I interferon
PyEBL: *P. yoelii* erythrocyte binding-like protein
SNPs: Single nucleotide polymorphisms
HGAP: Hierarchical genome assembly process
CCS: Circular consensus sequencing
AED: Annotation edit distance
pi: Post infection
PcEMA1: *P. chabaudi* erythrocyte membrane protein 1
MMEJ: Microhomology-mediated end joining
BUSCO: Benchmarking universal single-copy orthologs
ETRAMPs: Early transcribed membrane proteins
VEP: Variant effect predictor
ISGs: Interferon-stimulated genes
*pir*: Interspersed repeat
*yir*: *Yoelii* interspersed repeats
iRBCs: iRed blood cells
QC: Quality control
HMMs: Hidden markov models
LCB: Co-linear block
