# Supplementary material for: Genome sequence, transcriptome, and annotation of rodent malaria parasite Plasmodium yoelii nigeriensis N67
Source: BMC Genomics. 2021 Apr 26;22:303. doi: 10.1186/s12864-021-07555-9 (PMC8072299; doi:10.1186/s12864-021-07555-9)
Supplement: Supplementary file 1 — Additional file 1: Figure S1. Strategies of genome assembly and annotation, and plot of contig length distributions of the Plasmodium y. yoelii N67 parasite genome assembly. a, Diagram illustrating the processes of aligning N67 CCS reads (A) and contigs (B) to the 17X genomes. HGAP, Hierarchical genome assembly process; CCS read, circular consensus sequencing read. b, Plot of contig length distribution. The X-axis is percentage of the contigs with lengths (base pair) greater than the values indicated on the Y-axis. Figure S2. Clustering of protein sequences from the Plasmodium y. nigeriensis N67-specific orthogroups and those that are not assigned to any orthogroup. The predicted protein sequences were aligned using ClustalW algorithm and clustered using procedures described in the Methods section. a, Fam-A/B proteins; b, YIR proteins (group 1); c, YIR proteins (group 2). Only bootstrap values higher than 70% are shown. [file 12864_2021_7555_MOESM1_ESM.zip › Supplementalry information.docx]

**Genome sequence, transcriptome, and annotation of rodent malaria parasite *Plasmodium yoelii nigeriensis* N67**

Cui Zhang^1*^, Cihan Oguz^2*^, Sue Huse^2^, Lu Xia^1,3^, Jian Wu^1^, Yu-Chih Peng^1^, Margaret Smith^1^, Jack Chen^4^, Carole A. Long^1^, Justin Lack^2^, and Xin-zhuan Su^1#^

*^1^Malaria Functional Genomics Section, Laboratory of Malaria and Vector Research, National Institute of Allergy and Infectious Disease, National Institutes of Health, Bethesda, MD 20892-8132, USA;*

*^2^NIAID Collaborative Bioinformatics Resource (NCBR) and Advanced Biomedical Computational Science,, Frederick National Laboratory for Cancer Research, Leidos Biomedical Research, Inc., Frederick, MD 21702, USA*

*^3^State Key Laboratory of Medical Genetics, Xiangya School of Medicine, Central South University, Changsha, Hunan 410078, The People’s Republic of China.*

*^4^The NCI sequencing facility, 8560 Progress Drive, Room 3007, Frederick Md 21701, USA*

**Supplementary information**

**Additional file 1. Fig. S1-2.**


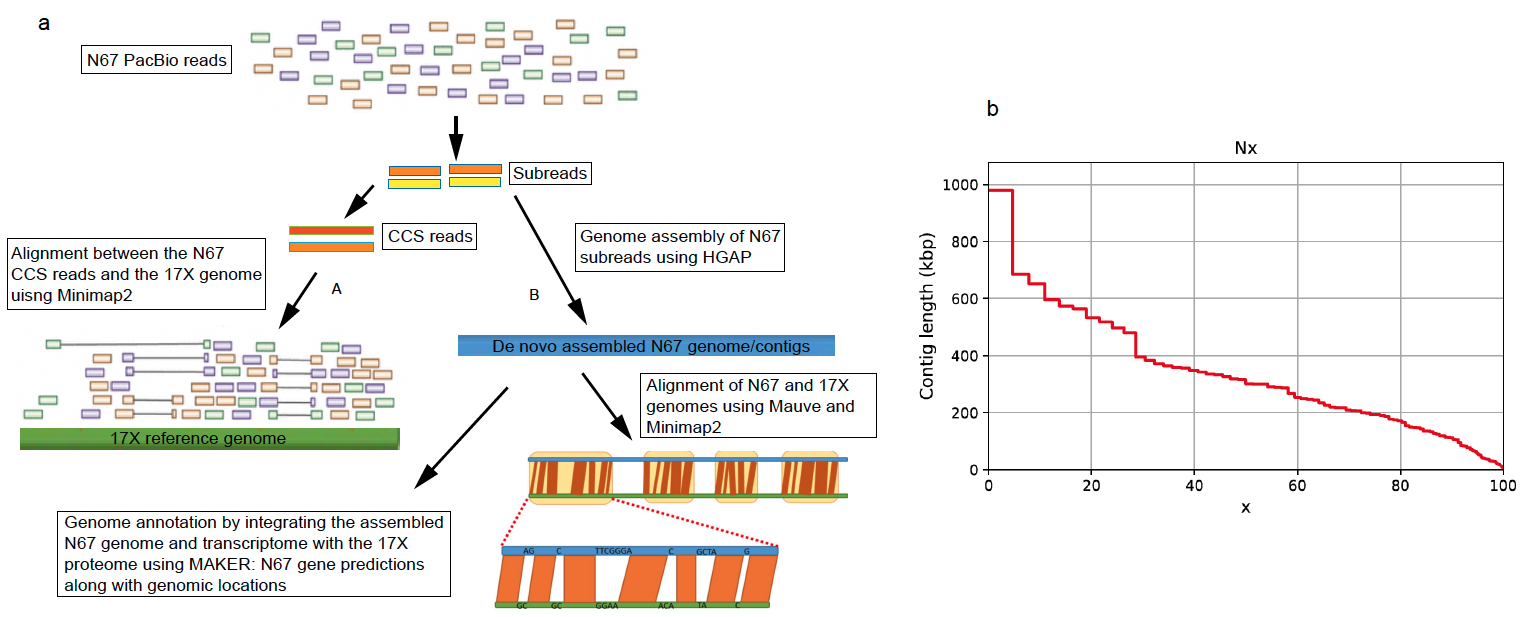


**Fig. S1**. Strategies of genome assembly and annotation, and plot of contig length distributions of the *Plasmodium y. yoelii* N67 parasite genome assembly. **a**, Diagram illustrating the processes of aligning N67 CCS reads (A) and contigs (B) to the 17X genomes. HGAP, Hierarchical genome assembly process; CCS read, circular consensus sequencing read. **b**, Plot of contig length distribution. The X-axis is percentage of the contigs with lengths (base pair) greater than the values indicated on the Y-axis.


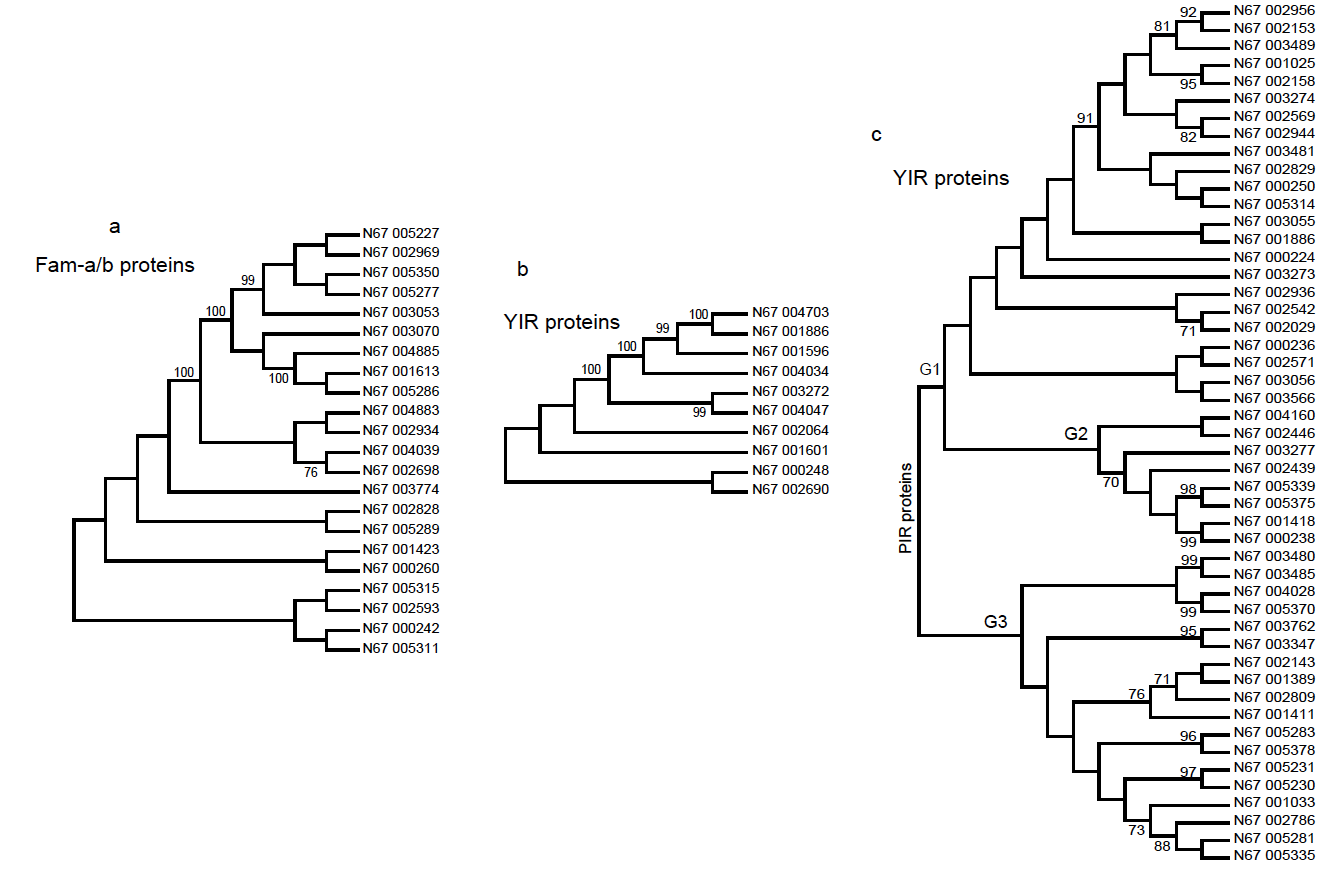


**Fig. S2**. Clustering of protein sequences from the *Plasmodium y. nigeriensis* N67-specific orthogroups and those that are not assigned to any orthogroup. The predicted protein sequences were aligned using ClustalW algorithm and clustered using procedures described in the Methods section. **a**, Fam-A/B proteins; **b**, YIR proteins (group 1); **c**, YIR proteins (group 2). Only bootstrap values higher than 70% are shown.

**Additional file 2. Table S1.**

**Additional file 3. Table S2.**

**Additional file 4. Table S3.**

**Additional file 5. Table S4.**

**Additional file 6. Table S5.**

**Additional file 7. Table S6.**

**Additional file 8. Table S7.**

**Additional file 9. Table S8.**

**Additional file 10. Table S9.**

**Additional file 11. Table S10.**

**Additional file 12. Table S11.**
