# Supplementary figures and images for: Genome sequence, transcriptome, and annotation of rodent malaria parasite Plasmodium yoelii nigeriensis N67
Source: BMC Genomics. 2021 Apr 26;22:303. doi: 10.1186/s12864-021-07555-9 (PMC8072299; doi:10.1186/s12864-021-07555-9)

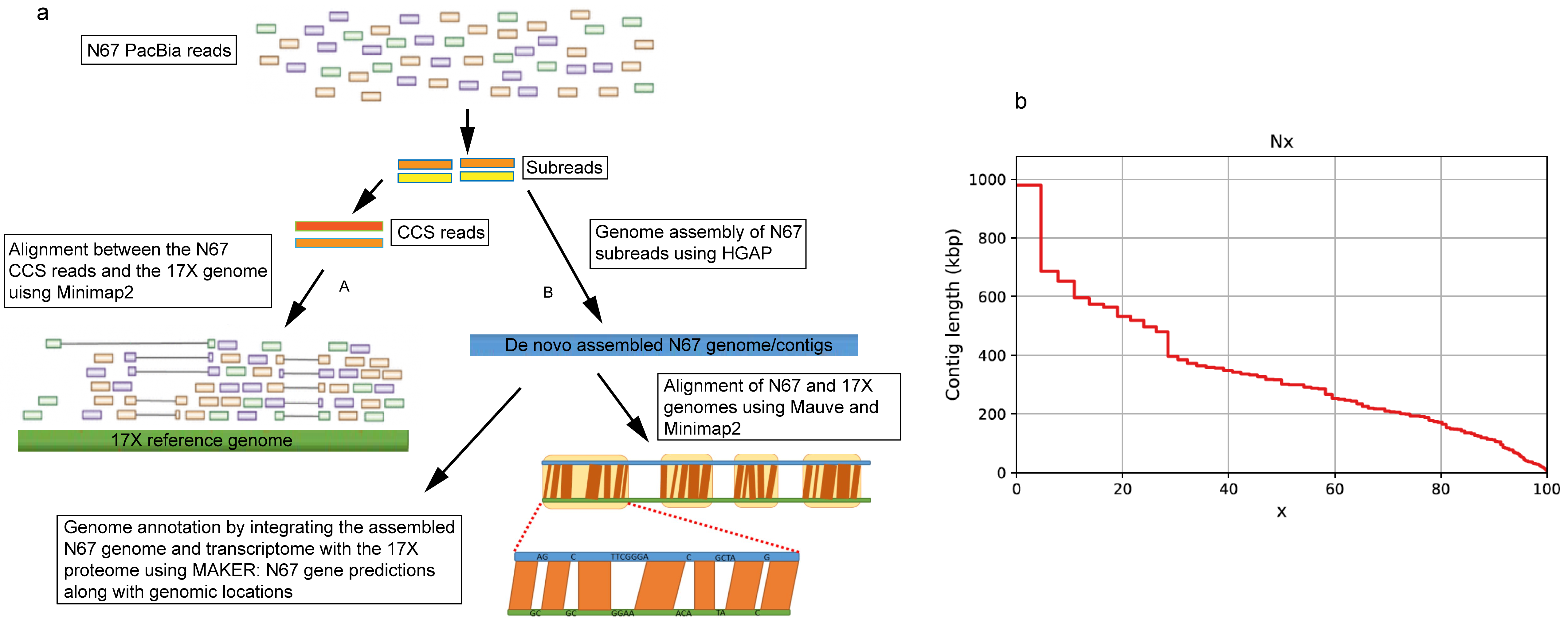

Supplement: Supplementary file 1 — Additional file 1: Figure S1. Strategies of genome assembly and annotation, and plot of contig length distributions of the Plasmodium y. yoelii N67 parasite genome assembly. a, Diagram illustrating the processes of aligning N67 CCS reads (A) and contigs (B) to the 17X genomes. HGAP, Hierarchical genome assembly process; CCS read, circular consensus sequencing read. b, Plot of contig length distribution. The X-axis is percentage of the contigs with lengths (base pair) greater than the values indicated on the Y-axis. Figure S2. Clustering of protein sequences from the Plasmodium y. nigeriensis N67-specific orthogroups and those that are not assigned to any orthogroup. The predicted protein sequences were aligned using ClustalW algorithm and clustered using procedures described in the Methods section. a, Fam-A/B proteins; b, YIR proteins (group 1); c, YIR proteins (group 2). Only bootstrap values higher than 70% are shown. [file 12864_2021_7555_MOESM1_ESM.zip › Figure S1.tif]

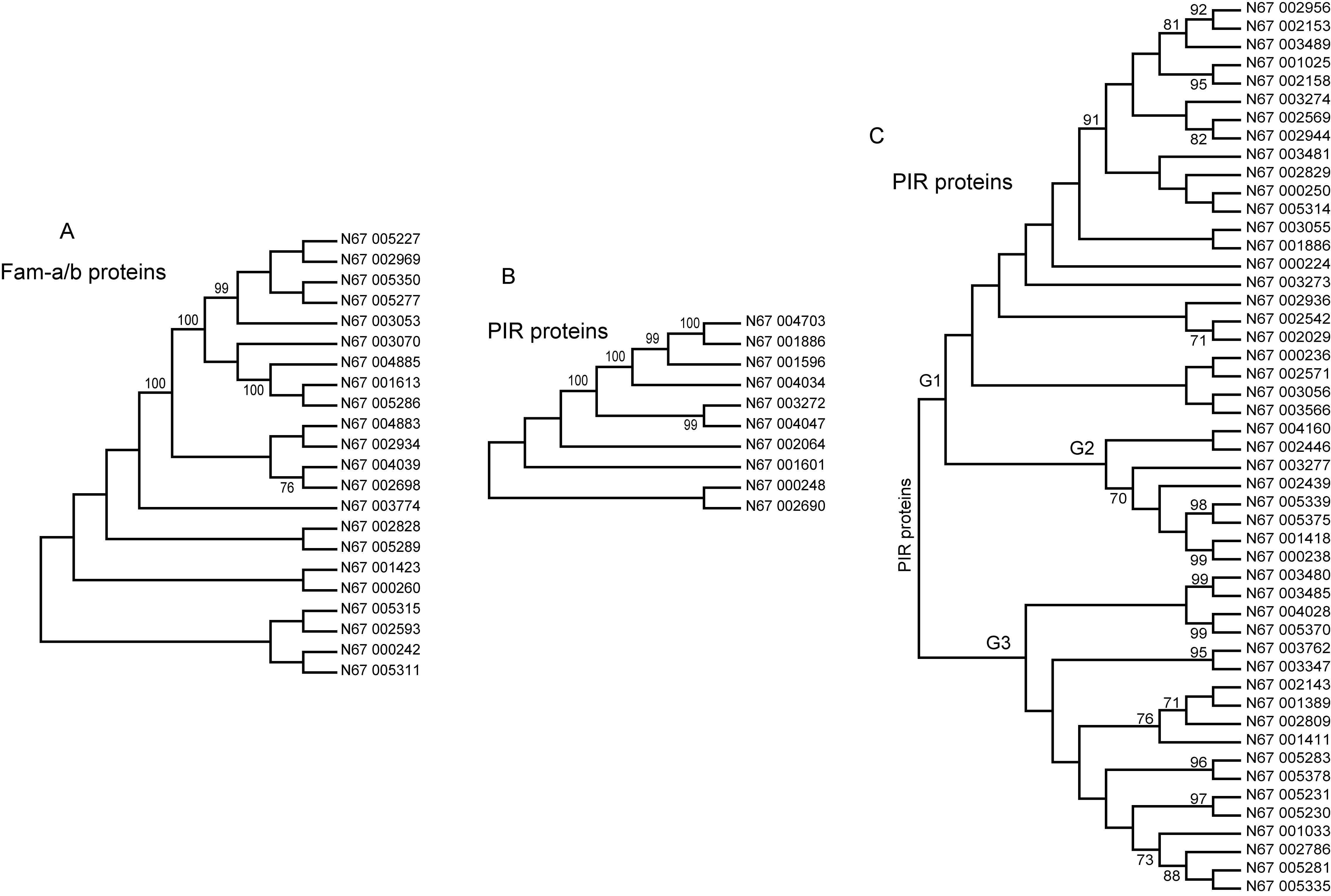

Supplement: Supplementary file 1 — Additional file 1: Figure S1. Strategies of genome assembly and annotation, and plot of contig length distributions of the Plasmodium y. yoelii N67 parasite genome assembly. a, Diagram illustrating the processes of aligning N67 CCS reads (A) and contigs (B) to the 17X genomes. HGAP, Hierarchical genome assembly process; CCS read, circular consensus sequencing read. b, Plot of contig length distribution. The X-axis is percentage of the contigs with lengths (base pair) greater than the values indicated on the Y-axis. Figure S2. Clustering of protein sequences from the Plasmodium y. nigeriensis N67-specific orthogroups and those that are not assigned to any orthogroup. The predicted protein sequences were aligned using ClustalW algorithm and clustered using procedures described in the Methods section. a, Fam-A/B proteins; b, YIR proteins (group 1); c, YIR proteins (group 2). Only bootstrap values higher than 70% are shown. [file 12864_2021_7555_MOESM1_ESM.zip › Figure S2.tif]
